# Poisoning by Opium

**Published:** 1869-12

**Authors:** J. E. O’Brien

**Affiliations:** Phillipsburg, N. J.


Article XI. — *Poisoning by Opium.* By J. E. O'Brien, M.D., Phillipsburg, N. J.

Appropos of opium and belladonna, I would offer the following case:

Emmy C., aged 2 years, found and swallowed thirteen pills of gum opium which had been prepared for the use of a neighboring opium eater's child. Each pill contained about one quarter of a grain.

Saw the little patient ten hours afterward. She was in stupor, respiration feeble and irregular, pulse slow and full, twitching of the tendons, pupils contracted to a point, tongue swollen and thrust between the lips, and trembling.

Obtained emesis by sulphate of zinc and ipecac. Used sinapisms to feet and gave coffee to drink.

In two hours no improvement. No irritant effect from the mustard. Gave a quarter grain ext. belladonna. In twenty minutes, respiration, from being almost gasping, became free and regular. Gave another quarter grain in half an hour, which was followed in an hour by the little girl getting up and asking for bread. She remained awake half an hour talking to her mother, then slept naturally until morning, when she appeared perfectly bright and fresh, and without debility.

*Theory.*—The opium had induced congestion of the nerve centres. The belladonna stimulated tissue change in them, by which the extra material was taken up and the capillaries relieved.

The opium eatress wishes to reform. Shall try the K. Br. brought for her to step down upon. Her children, she says, cry for their narcotic as soon as born. She weans them at about five years of age.

